# An Optimal Lysis Time Maximizes Bacteriophage Fitness in Quasi-Continuous Culture

**DOI:** 10.1128/mbio.03593-21

**Published:** 2022-04-25

**Authors:** Sherin Kannoly, Abhyudai Singh, John J. Dennehy

**Affiliations:** a Biology Department, Queens College of The City University of New York, New York City, New York, USA; b Department of Electrical and Computer Engineering, University of Delaware, Newark, Delaware, USA; c The Graduate Center of the City University of New York, New York City, New York, USA; University of Pittsburgh

**Keywords:** adaptation, bacteriophage lambda, event timing, holin, life history traits, optimization, timing of reproduction

## Abstract

Optimality models have a checkered history in evolutionary biology. While optimality models have been successful in providing valuable insight into the evolution of a wide variety of biological traits, a common objection is that optimality models are overly simplistic and ignore organismal genetics. We revisit evolutionary optimization in the context of a major bacteriophage life history trait, lysis time. Lysis time refers to the period spanning phage infection of a host cell and its lysis, whereupon phage progenies are released. Lysis time, therefore, directly determines phage fecundity assuming progeny assembly does not exhaust host resources prior to lysis. Noting that previous tests of lysis time optimality rely on batch culture, we implemented a quasi-continuous culture system to observe productivity of a panel of isogenic phage λ genotypes differing in lysis time. We report that under our experimental conditions, λ phage productivity is maximized around optimal lysis times ranging from 60 to 100 min, and λ wildtype strain falls within this range. It would appear that natural selection on phage λ lysis time uncovered a set of genetic solutions that optimized progeny production in its ecological milieu relative to alternative genotypes. We discuss this finding in light of recent results that lysis time variation is also minimized in the strains with lysis times closer to the λ wild-type strain.

## INTRODUCTION

Evolutionary biologists have long been interested in the power of natural selection to refine biological adaptations (1–4). Generally, views on the power of natural selection fall into two camps, which are not mutually exclusive: one emphasizing the ability of natural selection to optimize biological traits (2, 5, 6) and the other stressing the limitations of natural selection in face of evolutionary tradeoffs and genetic details (3, 4, 7) affecting phenotypic traits. Despite the widespread popularity of optimality theory, there exist relatively few experimental demonstrations of optimality in nature (7–9). Nonetheless, optimality models have provided valuable insights into biological traits ranging from the genetic code to animal behavior (5, 10–16) and so they are a crucial component of modern evolutionary theory. Here we attempt to address the disconnect between the perceived value of optimality theory in generating evolutionary insight with the relative paucity of experimental evidence for this foundational principle.

Because of their relative simplicity and amenability to experimental evolution, microorganisms, including bacteriophages (phages), have been popular model organisms to test predictions regarding optimality theory (9). For example, numerous studies employing phages have applied optimization principles to theoretically predict how a major phage life history trait, lysis time, will evolve in response to changing ecological conditions (17–24). In phages, lysis time determines the number of offspring produced by a single phage (i.e., burst size) because intracellular progeny assembly is generally a linear function with respect to time (25, 26). Therefore, any deviation from the optimum lysis time should alter a phage’s reproductive output, leaving the phage less fit than an optimum genotype in a given environment. As such, a common experiment is to experimentally alter a phage’s lysis time and observe whether it will re-evolve to a theoretically predicted optimum (27–29).

However, despite clear predictions, experimental tests using phages have generally failed to confirm theoretical predictions. Phages T7, ST-1, and ΦX174 with theoretically predicted suboptimal lysis times generally failed to evolve predicted optimal lysis times (28, 29). While the evolution of T7 phage with a deleted lysin gene qualitatively supported optimality predictions, it was noted that abolition of the lysis function did not extend the latent period, thus had no effect on the expected trade-off between latent period and burst size (28). ST-1 qualitatively, but not quantitatively, adapted as predicted by modeling, and ΦX174 failed to adapt at all (29). Several authors have theoretically predicted that at higher host densities, phages can evolve to grow faster by reducing the latent period (17–20). Experimental evolution of phage RB69 did result in selection for shorter lysis time mutants in high host density cultures, but it is unclear whether the lysis time of these mutants were optimal for the experimental conditions (27). A study employing isogenic lambda phages with different lysis times found the model predictions for both the optimum and fitness to be different from the experimental estimations (26).

However, we note that these studies used batch cultures to examine host-phage interactions, and phage fitnesses were estimated using absolute growth rates. These conditions may not effectively mimic the kinds of conditions experienced by phages in their natural habitats. To revisit the question of phage optimal lysis time, we employed a different approach, which was inspired by Bonachela et al. (22), who modeled host-virus interactions in natural environments in terms of steady state conditions, such as those seen in continuous culture systems. A two-stage chemostat is an ideal continuous culture system that enables phage evolution by keeping host constant. In the first stage, a chemostat receives a flow of fresh media to maintain a continuous culture of bacteria. Fresh host bacteria from the first chemostat are fed into a second chemostat containing a population of phages, which reduces the possibility of phage-host coevolution. Both host cells and phages are washed out from the second chemostat at a specified rate. This system can mimic steady states that exist in natural environments, such as the gastrointestinal tract where peristaltic movements can maintain a continuous flow.

Lysis is the last step in the infectious cycle of lytic bacteriophage, resulting in the release of virions. Lysis timing in many phages is controlled by a single protein, holin, which accumulates in the cell membrane up to a genetically determined time, when it nucleates generating membrane-permeabilizing holes (30, 31). The lysis timing is subject to variation arising from stochastic gene expression and other dynamic intracellular processes (32, 33). In a previous study, we measured the cell-to-cell variation or “noise” in lysis timing for wild type λ and several holin mutants that showed a wide range of lysis times (34). Intriguingly, we observed that the wild type as well as mutants that lysed around the same time as the wild type showed reduced noise in lysis time. This led us to the question: Is there a biological significance to minimizing the noise in lysis timing? What are the growth conditions under which lysis time becomes an important factor in maintaining phage populations? We hypothesized that under conditions of constant dilution, where cells and phages are regularly washed out, lysis time will play an important role in maintaining phage populations.

In this study, we maintained phage λ and its host, Escherichia coli, under quasi-steady state conditions. To initiate experiments, λ holin mutants differing in their lysis times (32–34) were added to exponentially replicating batch cultures of E. coli. After a short period of growth, λ phages were filtered from host cells, and a small fraction was transferred into a fresh exponential culture. This transfer was repeated serially for a total of five transfers. At no time did these phages experience stationary phase host cells during the experiment. In principle, differences in lysis times should result in differences in phage productivity (26, 35). We predicted that there exists an optimum for lysis time that maximizes phage production under these quasi-steady state conditions. By contrast, strains with suboptimal lysis times should show declining phage populations in this experimental context. Our results show that phage production in these quasi-steady state conditions is maximized at lysis times ranging from 60 to 100 min, and λ wild-type strain falls within this range. These results suggest a range of optimum lysis times that maximizes phage progeny production under a given set of growth conditions.

## RESULTS AND DISCUSSION

Ideally, this study would be performed using actual chemostats, but we choose not to pursue this option because it would limit the number of holin mutants and degree of replication that we could easily manage. Moreover, it is well known that E. coli growth is consistent (i.e., in a steady state) if exponential growth conditions are maintained (36) so, in physiological terms, E. coli growth is not inherently different between chemostats and our quasi-steady state transfers. Such a steady state achieved by introducing fresh batch cultures ensures a balanced exponential phase throughout the transfers. The cells in exponential phase are in the same physiological state characterized by relatively similar cell volumes and macromolecular composition. This aspect ensures that the growth parameters such as cell density (number of cells per milliliter) and the concentration of key macromolecules such as proteins, RNA, DNA, etc., increase exponentially with precisely the same doubling time (37). It is important to note that, unlike previous studies, our study does not aim to explore evolution of an optimum lysis time because of phenotypic adaptation. Instead, we aim to test the productivity (i.e., total phage reproduction during a fixed growth period) of genotypes varying in their lysis times in a dynamic environment that is subject to constant dilution or wash out. In other words, we aim to explore short-term ecological interactions between a phage and its host in a dynamic environment, which may have long-term evolutionary implications. To this end, the experimental protocol was designed to mimic natural conditions where both phages and cells would be washed out of a productive habitat at regular intervals.

**FIG 1:**
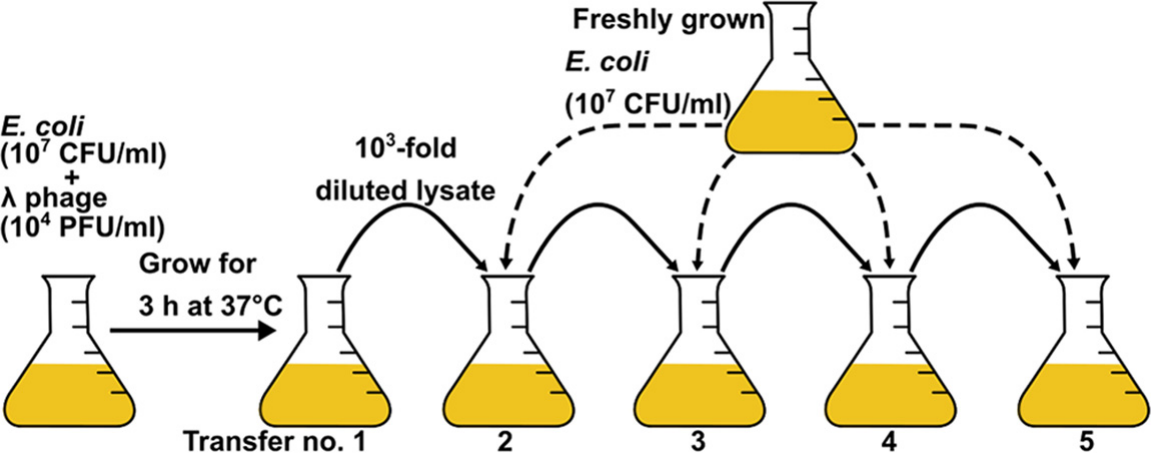
Serial transfers. For serial transfers, λ phage strains were cocultured with exponentially growing host cells, filtered to separate phages, and diluted 1,000-fold to start the next transfer with freshly growing cultures. After every transfer, the filtered phage lysates were titered using plaque assays.

The transfer conditions including the use of fresh cultures to initiate each subsequent transfer was optimized to maintain a quasi-steady state. These conditions were determined on the basis of pilot experiments using the wild-type strain (see Materials and Methods and Fig. 1) in which productivity was observed to steadily increase and then peak at the fifth transfer (Fig. S1). It is important to note that higher multiplicity of infections (MOIs) in later transfers can increase the chances of multiple infections per cell and faster host depletion, both lowering phage productivity (Fig. S1). The mean lysis time for wild-type strain is approximately 1 h. Each transfer lasting for 3 h would thus result in three generations. Therefore, a total of five serial transfers would result in approximately 15 phage generations. After 15 generations, it is possible that phage adaptation would begin to impact overall phage productivity. Therefore, we decided to use the productivity at the end of the fifth transfer for comparisons among different strains.

10.1128/mbio.03593-21.1 FIG S1 Serial transfers for wild type λ. Phage titers for three independent replicates consisting of a total of six transfers each. Error bars, mean ± SEM. Download FIG S1, TIF file, 0.1 MB. Copyright © 2022 Kannoly et al. 2022 Kannoly et al. https://creativecommons.org/licenses/by/4.0/ This content is distributed under the terms of the Creative Commons Attribution 4.0 International license.

**FIG 2:**
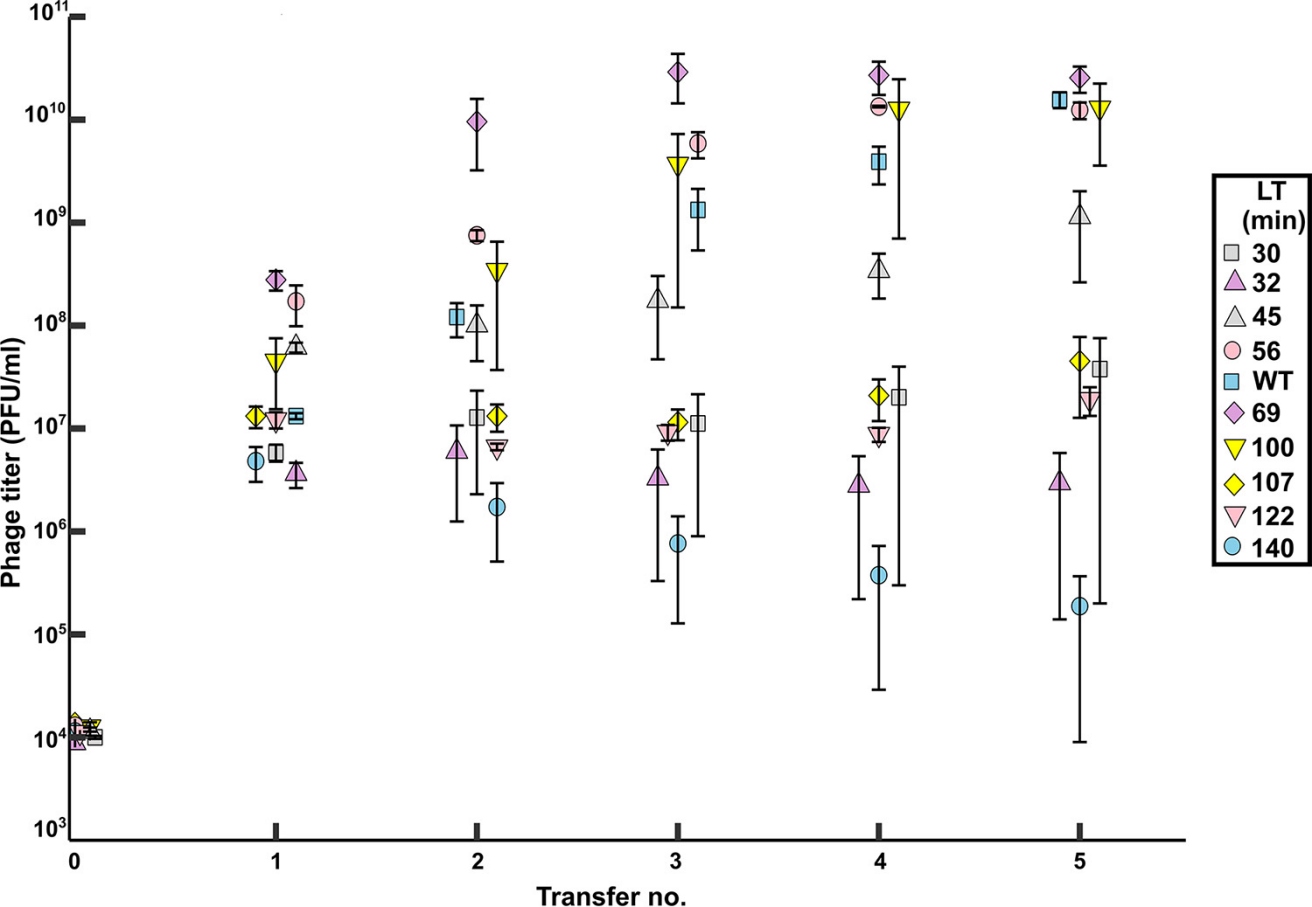
Titers of λ strains with different lysis times (LT) after each transfer. Lysis times are from single-cell estimates as reported in Kannoly et al. (34). WT lysis time = 63 min. Error bars, mean ± SEM.

**FIG 3:**
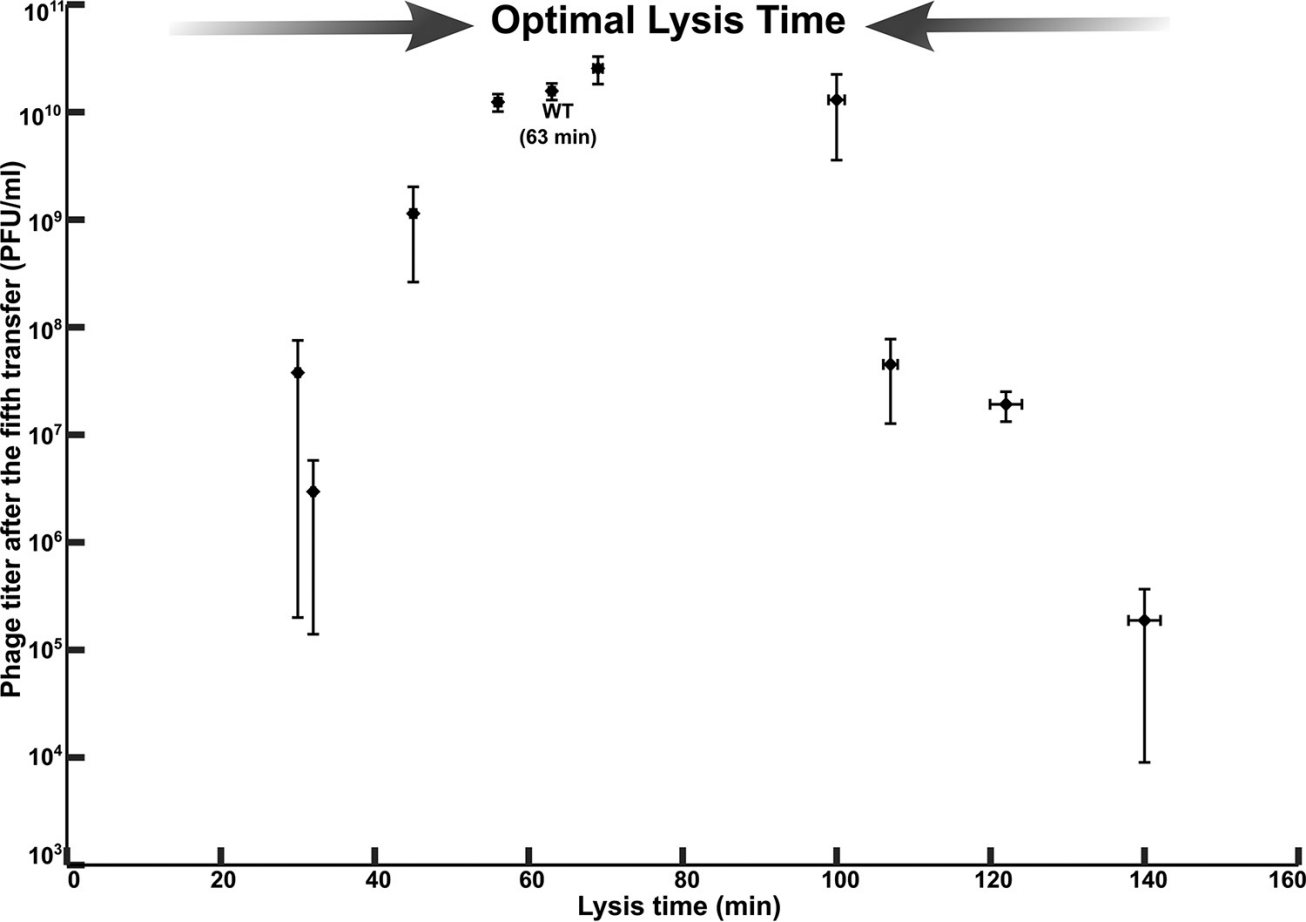
Titers of λ strains with different lysis times after the fifth transfer. Titers increase as the lysis time approaches the optimal range. Error bars, mean ± SEM.

Five serial transfers were completed in triplicates for each phage genotype, and the phage titers (i.e., phage concentrations) were obtained after every transfer (Fig. 1). Fig. 2 shows phage titers after every transfer for all the strains. The zero transfer represents the starting input titer for each strain, which was approximately 10^4^ PFU/mL. The subsequent transfers represent the phage titers at the end of each 3 h growth cycle of infecting fresh hosts. Host cell densities and physiological states have been known to play an important role in phage-host growth dynamics. In our experimental set up, we maintained a constant phage dilution rate (1,000-fold) and starting host density (≈10^7^ CFU/mL) at every transfer. Reaching a density of 10^7^ PFU/mL after the first transfer is critical to maintain the productivity at 10^7^ PFU/mL till the end of the fifth transfer. This is because a 1,000-fold dilution used to start the second transfer lowers the phage titer to the density that was used to initiate the first transfer (10^4^ PFU/mL). If dilutions are accurately performed, the strains that reach a critical threshold of 10^7^ PFU/mL can maintain this density throughout the transfer. After each transfer, the phage strains with lysis times between 60 to 100 min show increasing titers, which begin to plateau by the fifth transfer (Fig. 2). However, stochastic variations in diluting the phages can alter productivities in the subsequent transfers. In addition, if the adsorption process is stochastic, then early adsorption by a small proportion of phages will result in early release of progenies. This in turn would trigger a feedback cycle, increasing the overall productivity. Such fluctuations in productivities could have a compounding effect on the final productivity. This might explain the discrepancy observed in the strain with a lysis time of 30 min, which showed a higher final productivity than the strain with a lysis time of 32 min (Fig. 3). This also means that strains that show productivities higher than 10^7^ PFU/mL after the first transfer are more likely to show consistent increase in productivities in the subsequent transfers (Fig. 2). On the other hand, strains that can barely reach the critical density of 10^7^ PFU/mL after the first transfer show gradual decline in the later transfers (Fig. 2). This is because a 1,000-fold dilution in such cases means that the next transfer would begin with a density lower than 10^4^ PFU/mL. Thus, the steepest decline leading up to the fifth transfer was observed in the genotype with a lysis time of 140 min followed by the strain with a lysis time of 122 min (Fig. 2). Our data show that following five transfers, phage genotypes showing a broad range of lysis times spanning 60 to 100 min show increased phage productivities (Fig. 3). Interestingly, the phage λ wild-type strain falls within this range (Fig. 2 and 3). Outside this range, phage progeny production drops precipitously up to six orders of magnitude (Fig. 2). This outcome demonstrates the strong influence of lysis timing on the reproductive success of the phage.

Conceptually, phage production per generation depends on the burst size, which is positively correlated to the lysis time (26). The exact shape of this correlation is a matter of debate (38). A phage genotype with a short lysis time, and hence a low burst size, would be minimally productive over the experimental time span. Thus, for fast lysing phages, fewer phages are carried over to the next transfer. Conversely, one might intuitively assume that, for slow lysing phages, the phage productivity will be significantly higher. However, the delay in lysis traps a significant number of phages within cells, and these cells fail to be transferred to a fresh habitat (i.e., are washed out). Although delaying lysis increases the number of progenies, it also delays the generation time. This trade-off makes the optimum sensitive to environmental conditions such as dilution due to a constant flow rate. When the lysin gene was deleted in the T7 phage, the lysis time was shown to re-evolve to approach that of the wild type (39). However, the deletion did not greatly delay lysis because holin-mediated membrane permeabilization reduced protein synthesis, ceasing phage production, and thus eliminating the trade-off that is an important assumption of optimality models. In contrast, our holin mutants with significantly longer lysis times show continued phage production (40). Our experimental set up was sensitive to dilution due to a constant flow rate, and the strains with lysis times that deviate from the optimum range show reduced phage productivity over subsequent transfers (Fig. 2 and 3). This set up may more closely mimic the range of conditions experienced by the ancestors of E. coli and phage λ in the animal gut milieu. The objective of this study was to elucidate short term ecological dynamics that may be predictive of long-term evolutionary dynamics. However, such studies may not necessarily provide insights into evolutionary dynamics due to genetic constraints that may play important but hitherto unknown roles. This was observed in a study where optimal foraging by bacteriophage ΦX174 could predict the ecology but not the evolution of host specialization (41). We have no *a priori* basis to predict an optimum lysis time, but an optimum range is suggested from the observation that mutants with an intermediate range of lysis times had higher phage production than those with shorter and longer extremes.

Between transfers, the MOI for strains that showed higher productivities (for e.g., wild type or strain with lysis time 69 min) would shift to higher values when compared to the MOI values of strains with lower productivities (for e.g., strains with lysis time 30 min or 140 min in Fig. 2). Such increases in MOIs between the transfers can increase the chances of multiple infections per cell and faster host depletion, both lowering phage productivity. The effect of changing MOI within a transfer is exacerbated in strains with lysis times in the shorter and longer extremes. The strains with long lysis times will experience a relatively greater increase in MOI compared with the strains with very short lysis times. This bias would result in higher productivities for short lysing strains, which can be corrected only by keeping the MOI constant. This has implications in long-term evolution studies where it was shown that higher host densities (≥~10^7^ CFU/mL) tend to select for strains with short lysis times (27). Because we maintain host densities of ~10^7^ CFU/mL, our productivity measures most likely will not be significantly affected by the shifting MOI. However, our study looks at much smaller time scales, which is insufficient to observe the increase in frequency of adaptive mutations. With all else being equal, the holin mutants used in this study differ only in their lysis timing. In a previous study using these mutants, we demonstrated that holin accumulation thresholds generate precision in lysis timing. We showed that cell-to-cell variation or “noise” in lysis timing is reduced in mutants with lysis times closer to that of the wild type (34, 42). In the current study, mutants with lysis times closer to that of wild type showed increased productivity. Thus, noise in lysis timing appears to be negatively correlated to phage productivity. This suggests that holin mutations that lower noise may be favored by selection under conditions such as dilution due to a constant flow rate. This is in stark contrast to previous studies, which have suggested that mutations that increase lysis time variance would be favored by selection (38, 43). Baker et al. (38) used an experimental design where they used lysis gene mutants to study the effects of lysis time on burst size trait. Using a novel statistical framework, they showed significant heterogeneity in burst size, lysis time, and lysis time variance. Bull et al. (43) have used simulated transfer protocols similar to our study to model the effect of lysis time variance and cell growth. In contrast, our study measured the effect of lysis time on overall phage productivity under conditions of constant dilution. It was hypothesized that since phage burst size increases linearly (25, 26), early lysis events would contribute more to population growth than late lysis events detract (38, 43). Interestingly, Storms et al. have reported that T4 phage productivity in a cell about to undergo cell division was almost three times greater than the productivity in a young, newly formed cell (44). We speculate that if the contribution of early lysis events to population growth is balanced by larger bursts in the later stages of infection, mutations that increase lysis time variance may not be favored after all (38).

Our results add additional support for the idea that life history traits evolve to optimal values. For a theory that many regard as a fundamental foundation to the principle of adaptation by natural selection, optimality theory has surprisingly little concrete experimental support. Much work has been conducted experimentally assessing the validity of optimality models with varying degrees of success (8, 27–29, 45–51). Part of the issue is that a common approach to testing for optimality is by disrupting an organismal phenotype through genetic manipulation and expecting the re-evolution of an optimized trait at a rather short timescale (e.g., 10 to 100 generations). Consequently, it is often suggested that optimality evolution has failed because of genetic details or constraints (20). So, instead of perturbing a system, our approach instead inverts this question to ask, given a suite of genotypes that produce a wide range of phenotypes for a fundamental life history trait, which genotypes produce the greatest number of offspring across several generations under a given set of growth conditions? The answer, as it turns out, is the genotypes that show an intermediate range and not shorter and longer extremes of a phenotype.

## MATERIALS AND METHODS

### Strains.

All bacteria and lysogen strains used in this study are listed in Table 1.

**TABLE 1:** All bacteria and lysogen strains used in this study are listed along with the genotype of the prophage’s holin gene and the source of the strain

Strain	Genotype	Lysis time (min)	Source
CGSC#: 6152^*a*^	E. coli MC4100 (λ-)	NA	55
JJD3	MC4100 (*λ cI857 S*)	63	26
JJD246	MC4100 (*λ cI857 S105_H7D_*)	69	34
JJD248	MC4100 (*λ cI857 S105_F94C_*)	56	34
JJD391	MC4100 (*λ cI857 S105_A16G/K92Q_*)	140	34
JJD404	MC4100 (*λ cI857 S105_I21V_*)	30	34
JJD405	MC4100 (*λ cI857 S105_V45G_*)	32	34
JJD414	MC4100 (*λ cI857 S105_L90I_*)	45	34
JJD423	MC4100 (*λ cI857 S105_F27Y_*)	122	34
JJD432	MC4100 (*λ cI857 S105_S89W_*)	107	34
JJD436	MC4100 (*λ cI857 S105_K92N_*)	100	34

a Coli Genetic Stock Center.

### Plaque assays.

The plaque assays were designed for minimizing variation in plaque size and enabling plaque counting. The E. coli strain MC4100 was grown overnight in tryptone broth (TB) broth (5 g NaCl and 10 g tryptone in 1 L water) plus 0.2% maltose at 37°C. The overnight culture was diluted with equal volume of TB + maltose and grown for another 1.5 h. Then, 100 μL of these cells were mixed with appropriately diluted phage lysates and incubated at room temperature for 20 min to allow pre-adsorption. This mixture was then added to 2.5 mL of molten H-top agar (52), gently vortexed, and overlaid onto freshly prepared plates containing 35 mL LB agar (10 g NaCl, 10g Tryptone, 5 g yeast extract, and 15 g agar in 1 L water). The plates were then incubated at 37°C and plaques were counted after 18 to 22 h. Each assay was triplicated to estimate the average PFU per milliliters (PFU/mL).

### Thermal induction of lysogens to obtain phage lysates.

Lysogens were grown overnight in LB media at the permissive temperature of 30°C. Overnight cultures were diluted 100-fold and grown in a 30°C shaking incubator till an OD_600_ of 0.3 to 0.4 was reached. For heat induction, the cultures were transferred to a 42°C shaking water bath for 20 min. Following induction, the cultures were transferred to a 37°C shaking incubator until lysis. The phage lysates were filtered and titered using plaque assay.

### Single-cell lysis time determination.

To study the effect of lysis time on maintaining phage populations that undergo constant dilution, we employed a panel of λ phages harboring mutations in the holin gene *S*, which delay or hasten the lysis event. First, we used microscopy to visualize and record the lysis events of 100 to 200 individual cells for each holin mutant (34). The cells used for this purpose are lysogens that harbor λ phage genomes and can be heat induced to initiate the lytic cycle. When compared with lysis time estimations using either one-step growth curves (using free phages) or decline in turbidity (for lysogens), both of which use the shortest time period possible, microscopy allows a more accurate estimation of mean lysis times measured from the start of lytic induction to the point of cell lysis. We induced the lytic cycle in lysogens and recorded the lysis times of single cells to estimate the mean lysis time. The protocol for determining single-cell lysis times has been described previously (32). Briefly, a 200-μL aliquot of exponentially growing culture (OD_600_ = 0.3 to 0.4) of lysogens was chemically fixed to a 22-mm square glass coverslip, which was pretreated with 0.01% poly-L-lysine (mol. wt. 150 K to 300 K; Millipore Sigma, St. Louis, MO) at room temperature for 10 min. Using this coverslip, a perfusion chamber (RC-21B, Warner Instruments, New Haven, CT) was assembled and immediately placed on a heated platform (PM2; Warner Instruments, New Haven, CT). The heated platform was mounted on an inverted microscope stage (TS100, Nikon, Melville, NY), and the perfusion chamber was infused with heated LB at 30°C (Inline heater: SH-27B, dual channel heating controller: TC-344B; Warner Instruments, New Haven, CT). The chamber temperature was elevated to 42°C for 20 min and thereafter maintained at 37°C until ~95% cells were lysed. Videos of cell lysis were recorded using an eyepiece camera (10× MiniVID™; LW Scientific, Norcross, GA). The lysis times of individual cells were visually determined using VLC media player. Lysis time was defined as the time required for a cell to disappear after the temperature was increased to 42°C. For each lysogen, an average of approximately 100 cells was used to calculate the lysis time.

### Serial transfers.

We used wild-type λ productivity as a benchmark for comparisons with the holin mutants. To this end, we performed pilot studies by initiating wild-type λ infections of host cells. Phage λ is known to lyse most infected cells in an exponentially growing culture with a small proportion of cells producing lysogens (53). However, a majority of infected cells under stationary phase conditions divide to produce lysogens. Lysogens cannot be infected by free phages and, therefore, lower phage productivity. To minimize the formation of lysogens, infections were initiated with exponentially growing cultures. A low MOI of 0.001 was used to start the first growth cycle. This minimizes multiple infections per cell and ensures multiple generations without depleting host cells to such an extent that the cell number becomes a limiting factor for phage growth. Moreover, at such a low MOI, there are 1,000 cells for each phage, thus reducing the adsorption time. After 3 h of infection, cell lysis outpaces growth for a short time, during which lysogenic events increase in frequency (54). After this point, the lysogenic cells grow exponentially and take over the culture until the stationary phase, which results in low phage productivity. Also, a 3 h infection allows at least three generations for wild-type λ such that an MOI ≤~ 1 is reached. Therefore, after 3 h of growth, the phages were purified, diluted 1,000-fold, and transferred to initiate the next round of infection with cultures containing the same concentration of fresh exponentially growing cells. The phage growth cycles were repeated for a total of five transfers with three independent replicates. Wild-type phage populations showed a decreasing trend and therefore an increased likelihood of extinction when a growth period of less than 3 h per transfer and/or a dilution rate greater than 1,000-fold were used (data not shown). To initiate the transfers, an E. coli (~10^7^ CFU/mL) culture growing exponentially in LB at 37°C in a shaking incubator (200 rpm) was infected with a phage lysate (~10^4^ PFU/mL). The infection could proceed for 3 h, after which phages were separated by filtration using a 0.2 μ syringe filter (Pall Corp.). The filtered lysate was diluted 1,000-fold to start the next transfer using freshly growing cells (~10^7^ CFU/mL). These steps were repeated four more times for a total of five serial transfers (Fig. 1). Plaque assays were performed at the end of each transfer to determine the titers of phage lysates. The transfers for all strains were triplicated.
