# Supplementary figures and images for: An Optimal Lysis Time Maximizes Bacteriophage Fitness in Quasi-Continuous Culture
Source: mBio. 2022 Apr 25;13(3):e03593-21. doi: 10.1128/mbio.03593-21 (PMC9239172; doi:10.1128/mbio.03593-21)

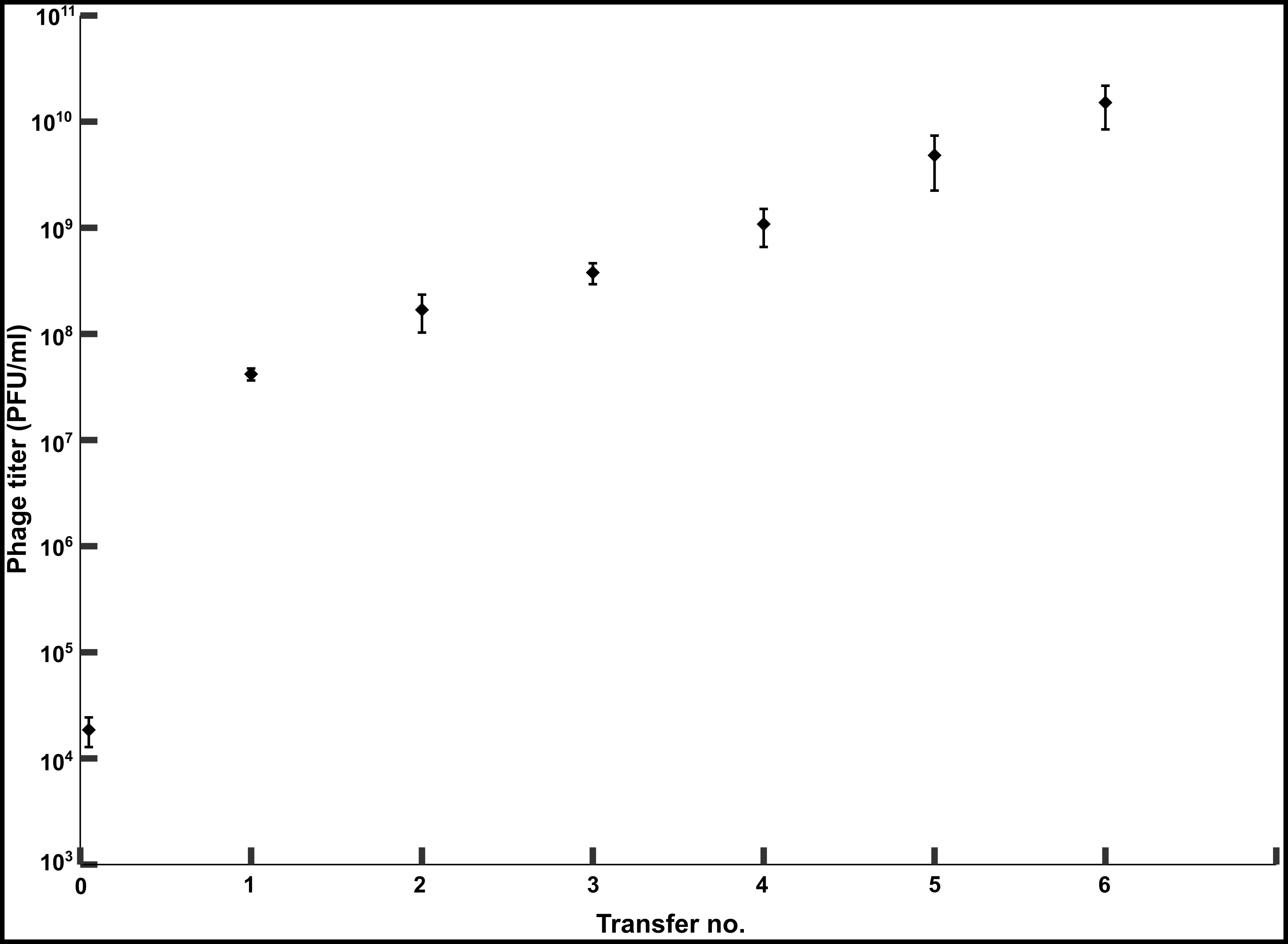

Supplement: FIG S1 [file mbio.03593-21-s0001.tif]
